# Navigating the *Nanoverse*: how emerging nanomaterials are transforming bioscience and society

**DOI:** 10.1039/d5na00940e

**Published:** 2026-04-20

**Authors:** Asia Saorin, Ahmed Subrati, Gloria Saorin, Giulia Yuri Moscatiello, Carmina Natale, Roger Bellido-Peralta, Giulia Cazzador, Mélina Guérin, Michele Crozzolin, Montserrat Vallet-Buisan, Anita Salmaso, Benjamin Punz, Sara Catalini, Annalisa Morelli, Alberto Martinez-Serra

**Affiliations:** a Department of Chemistry, Royal College of Surgeons in Ireland (RCSI) D02 YN77 Dublin Ireland; b Center for Cooperative Research in Biomaterials (CIC biomaGUNE), Basque Research and Technology Alliance (BRTA) Donostia San Sebastian 20014 Spain; c Department of Chemistry, University of Basel Mattenstrasse 24a, BPR-1096 4058 Basel Switzerland; d Department of Biochemistry and Molecular Pharmacology, Istituto di Ricerche Farmacologiche Mario Negri IRCCS Via Mario Negri 2 20156 Milan Italy; e National Graphene Institute, The University of Manchester Manchester UK; f Department of Molecular Sciences and Nanosystems, Ca' Foscari University of Venice Via Torino 155 Mestre 30172 Venezia Italy; g University of Angers, INSERM, CNRS, MINT, SFR ICAT 49000 Angers France; h Nuffield Department of Women's & Reproductive Health, Institute of Reproductive Sciences, University of Oxford Oxford OX3 9DU UK; i Department of Biosciences & Medical Biology, Paris Lodron University of Salzburg Hellbrunnerstrasse 34 5020 Salzburg Austria; j European Laboratory for Non-Linear Spectroscopy Via Nello Carrara 1, 50019 Sesto Fiorentino Florence Italy; k Department of Chemistry “Ugo Schiff”, University of Florence Via della Lastruccia, 3-13, 50019 Sesto Fiorentino Florence Italy; l Barcelona Supercomputing Center (BSC) Plaça Eusebi Güell 1-3 08034 Barcelona Spain alberto.martinez@bsc.es

## Abstract

The rapid expansion of nanomaterials research has given rise to the concept of the *Nanoverse*, a multidisciplinary shared domain where increasingly sophisticated nanoscale architectures address societal challenges while introducing new safety and sustainability questions. This review writes up recent advances across diverse nanomaterial classes, including carbon-based nanomaterials, two-dimensional membranes, chromopeptide nanoarchitectures, microfluidic drug delivery platforms, extracellular vesicles, and antimicrobial nanomaterials. We trace the evolution of nanomaterial definitions, noting the widening scope to include particles up to micrometer size, and highlight how unique physicochemical properties govern biological uptake, intracellular fate, and environmental behavior. Using micro- and nanoplastics as a case study, we emphasize the need for realistic exposure assessments, biomolecular corona characterization, and ethical models for toxicological analysis such as *C. elegans*. By integrating material science, biotechnology, toxicology, and environmental science, we map the *Nanoverse* as both a technological frontier and a regulatory challenge. We believe that realizing its full potential requires harmonized characterization protocols, scalable manufacturing, and sustainable design principles to ensure that transformative nanomaterial applications advance responsibly from the labs to society.

## Abstract 2

This review navigates the *Nanoverse*, an expanding dimension that connects emerging nanoscale materials with medicine, biosensing, and safety, highlighting how innovation, regulation, and ethics converge to guide nanotechnology for a better society.

## Graphical abstract

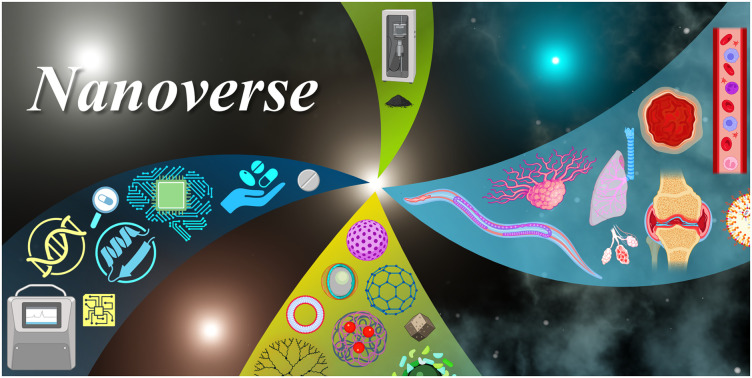

## 1 Introduction

The European Union originally defined nanomaterials (NMs) in 2011 as natural, incidental, or manufactured materials containing isolated, aggregated, or agglomerated particles, where 50% or more of them in the number-based size distribution have one or more external dimensions between 1–100 nm.^1^ This definition was updated on June 10, 2022, introducing stricter criteria: at least 50% of particles must meet one of the following conditions – (i) one or more external dimensions between 1–100 nm; (ii) elongated shape (*e.g.*, rod, fiber, tube) with two dimensions <1 nm and the third >100 nm; or (iii) plate-like shape with one dimension <1 nm and the other being >100 nm.^2^ This demonstrates that the concept of NMs is evolving along with the progress of NMs development and the increasing concern for their possible effect on human health and environment. Beside this definition, researchers nowadays commonly use the term NMs to refer to particles with size up to micrometer scale which can have a very wide range of composition, size and shape.^3^ This has led in 2015 to the use of the term ‘nanoParticle Zoo’ for describing the state of the field.^4^ However, in the last ten years, NMs and nanoparticles (NPs) have evolved and spread further moving from scientific curiosities into foundational elements of modern technology, shaping innovations across medicine, energy, electronics, and environmental science. Therefore, we enter what can be called the *Nanoverse*, a rapidly growing dimension where increasingly tailored NMs address societal challenges such as vaccine development and energy production, while also becoming widespread in everyday products, leading to their presence in the environment. Indeed, the unique physicochemical properties of NMs, arising from their size, surface area, quantum effects,^5^ and structural tunability,^6^ have unlocked previously unimaginable capabilities, from single-molecule biosensing to ultra-efficient energy storage.^7,8^ Emerging manufacturing strategies now combine precision molecular design with scalable, high-throughput production, focusing on multifunctional hybrids and engineered materials capable of addressing industrial, environmental, and biomedical challenges.^9–13^

**Fig. 1:**
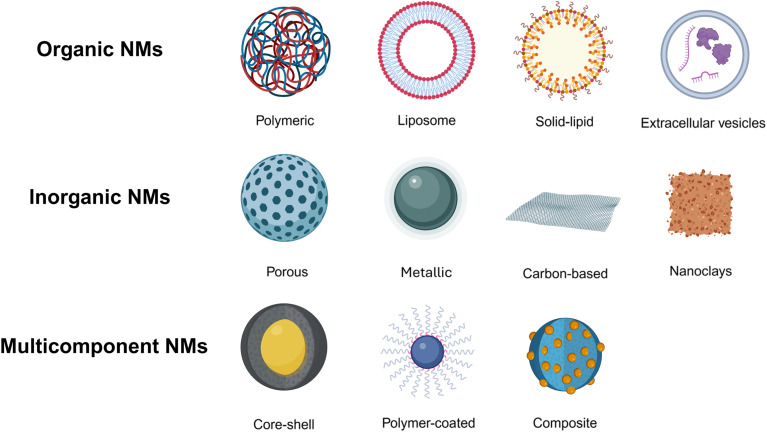
Classification of common NMs across different research fields by molecule nature: organic, inorganic, and multicomponent. Created with BioRender.com (https://www.biorender.com/).

Among the many classes of NMs, a broad distinction can be made between organic, inorganic, and multifunctional nanomaterials, each offering unique properties and application potential (Fig. 1). Inorganic NMs encompass a wide range of materials, among which carbon-based NMs (CBNs) such as graphene, carbon nanotubes, and carbon nanodots have demonstrated extraordinary versatility due to their exceptional electrical conductivity, mechanical strength, and functionalization potential.^14^ Simultaneously, two-dimensional (2D) materials have opened new frontiers in molecular separation,^15^ enabling precision filtration^16^ and environmentally responsive membranes.^17–19^ Organic NMs, including lipid- or polymer-based systems, have attracted increasing attention due to their biocompatibility and capacity for functional design. For example, chromopeptide nanoarchitectures, *i.e.* self-assembled structures combining optical and structural functionality, have emerged as intelligent platforms for light-driven diagnostics and therapies.^20^ Multifunctional nanomaterials are engineered systems that combine two or more distinct physicochemical, biological, or technological functionalities within a single nanoscale platform.^21,22^ Examples within the biomedical field include inhalable nanoformulations for respiratory and cardiovascular therapies,^23,24^ macrophage-targeting smart nanocarriers for precision medicine,^25,26^ extracellular vesicles as naturally derived platforms for targeted drug delivery,^27–29^ engineered NMs with antimicrobial properties capable of circumventing traditional resistance pathways,^30^ as well as the development of nanostructured theranostic platforms and wearable biosensors, offering enhanced sensitivity and specificity for real-time, personalized healthcare monitoring.^31^ All this influenced by technological advancements, such as the development of microfluidic platforms that enable continuous, scalable, and precisely controlled fabrication of nanomedicines with clinical relevance.^32,33^ However, the rise of these technologies brings along the critical need for rigorous toxicological assessment,^34^ including advanced characterization of NMs under realistic environmental and biological conditions,^35,36^ and investigations into the interactions of micro- and nanoplastics with living systems.^37,38^ Organisms like *Caenorhabditis elegans* (*C. elegans*) are increasingly recognized as ethically and biologically relevant *in vivo* models for evaluating the safety of NMs, offering insight into long-term exposure effects and sub-toxic responses that remain undetectable in simpler systems.^39^

Despite this momentum, the full potential of NMs remains largely untapped. Key challenges persist, including the lack of standardized descriptors, insufficient cross-domain data integration, and limited scalability of many promising lab-scale innovations.^40–43^ As we map the *Nanoverse*, it becomes essential not to only catalogue the materials and their functions but also to understand the systemic interconnections that allow nanotechnology to transform society at scale.

This review provides a cross-sectional analysis of emerging NMs and technology, focusing on their novel architectures, mechanisms of action, and translational applications. By synthesizing insights across material science, biotechnology, and toxicology, we aim to offer a comprehensive view of how these nano-scale constructs are driving macro-scale change—and what lies ahead in the continued exploration of the *Nanoverse*.

## 2 Transforming nanomaterial development for real-world impact

NM innovation is accelerating through the fusion of precision design and advanced manufacturing. Emerging approaches leverage molecular-level control, structural customization, and hybrid architectures to unlock new functionalities while enabling seamless translation from laboratory synthesis to industrial production. The next frontier lies in integrating adaptive design strategies with continuous, high throughput manufacturing platforms, paving the way for NMs tailored to meet the evolving demands of technology, medicine, and sustainable industries.

### 2.1 From novel carbon nanomaterials to breakthrough nanotechnologies

**Fig. 2:**
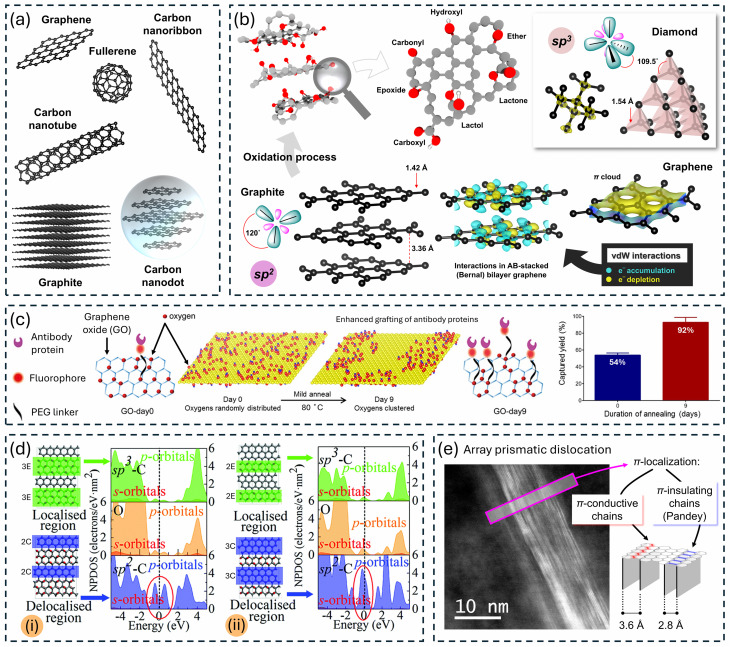
(a) Few examples of carbon-based materials. Structures were constructed from CIF files obtained from the Crystallography Open Database (COD)^74–76^ using Vesta.^77^ (b) Structures of graphene, graphite, graphite oxide, and diamond. Oxygen functional groups result from the oxidation of graphite endowing the layer material with multi-node hydrogen bonding. van der Waals (vdW) interactions are visualized for the graphene bilayer. Reproduced from Subrati *et al.*^78^ with permission from Wiley-VCH, Copyright (2024). (c) Oxygen-clustering in graphene oxide drives phase transformation separating graphitic sp^2^ and oxidized sp^3^ domains reshaping the percolation network on the carbon backbone reflected in reduced resistance while preserving the oxygen content. Antibody grafting is augmented in the oxygen-clustered derivative, and this leads to an almost two-fold improvement in cell capture efficiency. Reproduced from Bardhan *et al.*^70^ with permission from American Chemical Society, Copyright (2017). (d) Theoretical investigation on confinement of π-conjugated domains within oxidized domains on two models reveal strong impact on electronic nature of partially oxidized graphene. The two models vary in the width of the chains of epoxy groups (E, green) and the π-conjugated zigzag carbon chains (C, blue). DFT-calculated normalised projected density of states of partially oxidized graphene models: (i) semiconductor E3C2 and (ii) semi-metal E2C3. The carbon atoms enclosed in the blue and green rectangles contain delocalized and localized electrons, respectively. The partially delocalized sp^2^ carbons make up most of the electronic states near the Fermi level. Such states contribute immensely to quantum capacitance. These states facilitate partial charge transfer between electrolyte ions and redox centers *via* quantum capacitance leading to specific ionic adsorption and consequently fast reversible faradaic processes in partially oxidized graphene. Reproduced from Li *et al.*^79^ with permission from The Royal Society of Chemistry, Copyright (2017). (e) Experimental evidence of defects confining chains of electron delocalization (localized π-orbitals). Dark field – scanning transmission electron microscopy image of an array prismatic dislocation found in carbon ravioli of pyrolyzed graphite oxide framework. Abundance of such defects was increased by heating framework at 80 °C for 24 h in air prior to pyrolysis. This confinement of electron delocalizations augmented redox activity sizeably. Reproduced from Subrati *et al.*^71^ with permission from Elsevier, Copyright (2024).

Carbon has garnered the attention of various technological and biomedical fields with its exceptional chemical, physical, and electronic properties. Nanoscaling carbon has opened applied research horizons for abovementioned fields by introduction of carbon-based NMs (CBNs) such as graphene, fullerene, carbon nanotube, carbon nanodot, carbon onion, carbon nanodisk, carbon nanofiber, carbon quantum dot, activated carbon, nanodiamond, and carbon nanoribbon, as shown in Fig. 2(a and b).^44–50^ Superior electron mobility, tunable surface chemistry, high tensile strength, exceptional thermal and chemical stability, high porosity, tunable density and nature of defects, high surface area, and extremely low density are some of the properties that characterize CBNs and drive applied research in technological and biomedical fields. CBNs have been implemented in adsorption,^49^ drug delivery,^51^ wound healing,^52^ biomedical scaffolds,^53^ tissue engineering,^54^ prosthesis engineering,^55^ biosensing,^56^ photodynamic therapy,^57^ bioimaging,^47^ anticancer treatment,^51^ transistors,^58^ energy storage – such as batteries (lithium-ion batteries, lithium–sulfur ion batteries),^59^ (super-)capacitors,^60^ fuel cells^61^ and photovoltaics,^62^ quantum computing,^63^ spintronics,^64^ aviation fabrications,^65^ sensing,^66^ catalysis,^67^ and coating.^68^ Mention must be made of the possibility of discovery of new (sub-)classes of CBNs. Defect engineering in all CBNs holds great potentials for corresponding applications and is direct consequence of the tunable chemistry of CBNs.^69^ For instance, defective oxygen-functionalized domains were spatially clustered by action of mild thermal annealing (80 °C) of nanobodies-coupled graphene oxide to enhance cell capture from whole blood, as illustrated in Fig. 2(c).^70^ The ability of oxygen functionalities to migrate and cluster holds great potential not only in biological applications, but also in energy applications.^71^ Shaping the sp^2^ percolation network in all CBNs is theoretically predicted to significantly alter electronic properties, as demonstrated in Fig. 2(d). Defects in carbon materials, such as that in Fig. 2(e), can spatially and chemically localize sp^2^ bonds.^72^ Oxygen-clustering was evidenced to play a pivotal role in defining nitrogen doping configurations, which essentially impacts redox capabilities of nitrogen-doped carbon derivatives.^71^ Therefore, one can envisage that functionalization of CBNs with heteroatoms is not analogous to attaching stationary atoms. Heteroatoms, particularly oxygen, are mobile and their distribution can be tuned by action of mild thermal treatment. This step, often a post-processing step, is simple yet very deterministic and reflects orders of magnitude of potential new insights into already existing research utilizing CBNs. Thereby, there are still unexplored possibilities for useful and safe applications of CBNs. While many CBNs have been explored, their full potential remains untapped due to a lack of systematic archival reporting of their properties. The main obfuscated challenge from novel carbon NMs to breakthrough nanotechnologies is the establishment of universally accepted descriptors, numerical descriptors not only for physico-chemical characteristics, but also for application related parameters. Emerging efforts for grouping and read-across are already in place for few candidate CBNs.^73^ However, there is a pressing need for similar efforts for all explored CBNs. Established platforms that archive peer-reviewed research focusing on novel CBNs with corresponding physico-chemical descriptors and associated application related characteristics will facilitate human-readable comparisons as well as hastened accessibility, improved visibility, and recognition.

### 2.2 2D materials membrane design for molecular sieving purposes

**Fig. 3:**
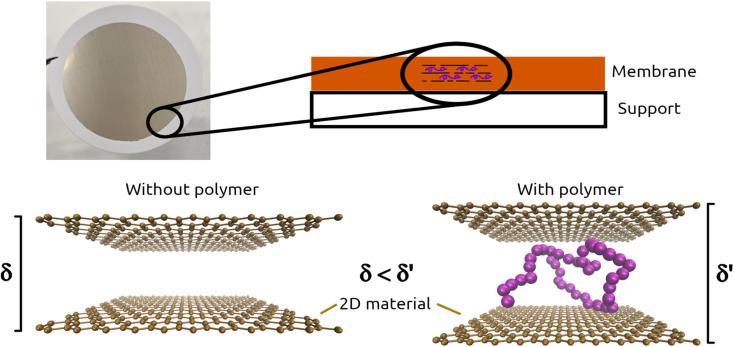
Schematics of a self-assembled 2D material membrane. Embedding polymeric chains between the nanoflakes conditions the properties of the final structure, *e.g.*, the interlayer space.

Current environmental issues range from atmospheric pollution to water contamination. There seem to be many solutions to these problems, but nanotechnology is constantly in the spotlight, more precisely 2D materials and their applications.^80^ There are multiple ways to use bidimensional structures to achieve different purposes such as drug delivery, anti-rust coatings or liquid filtration.^81,82^ The latter was discovered upon realising that self-assembled graphene oxide (GO) membranes were permeable to water but able to block other elements such as argon.^83^ In the past decade, efforts have been made to expand the range of materials that allow self-assembling membrane manufacture, *e.g.* graphene, graphene-related 2D materials (GR2M, like graphene oxide), transition metal dichalcogenides (TMD), MXenes, clays, MOFs, COFs and 2D heterostructures (alternate stacking of different 2D materials). A wide availability of materials translates into an also wide range of properties, which allows to devise all kinds of filtration and mixture separation procedures.^84–86^ Taking advantage of its laminar structure, stability and self-assembly properties, they can be used as filters to purify water,^87^ separate mixtures^88^ and even as molecular sieves.^89^ The performance and properties of membranes paired up with its facile manufacture allowed to scale up processes and are currently being tested for industrial applications.^90^ However, much research remains to be done, for example regarding molecular sieving properties. Membranes made from 2D materials assemble themselves by piling up nanoflakes, the filo pastry of nanotechnology, so to say. These structures have the capability to present different interlayer spaces (the distance between each layer),^91^ which in turn regulates the size of the molecules that can permeate. For example, water molecules flow with no constraint through graphene oxide (GO) structures, while contaminants can potentially be retained. However, what if we wanted water to be retained, while extracting larger molecules? Or how about extracting certain elements from a multi-component mixture? The solution seems to point towards polymers and how to appropriately fuse them with bidimensional materials. An easy and promising method is to cross-link polymeric chains to the layers of the membranes, thus keeping the laminar structure intact (Fig. 3).^92^ Using this assembly, we can force an interplay between the properties of both materials, obtaining new phenomena that are non-characteristic to any of them separately. This new type of structure could be the next step in the field of nanofluidics, as we can selectively tune the properties we desire in the resulting outcome. Composite membranes could be the answer to greener and more efficient biofuel manufacturing, less costly water filtering procedures or advances in the food industry. Be as it may, nanotechnology (and self-assembled membranes in this particular case) can lead the field further towards developments that make a difference.

### 2.3 Chromopeptide nanoarchitectures for the development of next-generation photonic devices

The emergence of NMs has marked a significant milestone in the development of various scientific fields, particularly medicine^93^ and photonics.^94^ Their impact stems from unique chemical and physical properties that arise at the nanoscale,^95^ enabling the creation of functional materials with capabilities not present in their monomeric forms.^96^ Within this context, supramolecular chemistry has proven to be a powerful bottom-up strategy for constructing highly organized, dynamic nanoarchitectures from molecular building blocks.^97–99^ These systems often exhibit emergent properties such as optical, magnetic, and electronic, resulting from precise molecular organization rather than individual component features.^97–99^ Notably, nanostructures assembled from small molecules can show intrinsic biological activity, even without incorporating additional bioactive agents.^100^ Among these, self-assembling peptides have attracted considerable interest for biomedical applications due to their ability to encode diverse bioactive functions.^95^ However, high production and purification costs often limit their practical use, prompting the search for short, cost-effective sequences that still retain self-assembling capacity.^95^ A prominent example is derived from amyloid beta (Aβ) peptide, associated with Alzheimer's disease. Efforts to identify minimal sequences responsible for Aβ′s self-assembly have highlighted the pentapeptide Lys-Leu-Val-Phe-Phe, recognized as the shortest natural l-peptide capable of forming amyloids in physiological conditions without modification.^101^ Earlier, in 2003, a breakthrough identified diphenylalanine (Phe–Phe) as a minimal motif that forms stable nanotubes in aqueous media.^102^ This foundational discovery led to the development of a new class of minimalistic peptide-based nanostructures.^103,104^ Advancing from this groundwork, researchers have begun exploring hybrid systems that combine peptide self-assembly with photonic functionality. At this intersection lie chromopeptide nanoarchitectures, *i.e.* self-assembled structures composed of peptides conjugated with chromophores. These materials offer molecular precision, tunable morphologies, and intrinsic biocompatibility, along with the capacity to tailor light–matter interactions.^20^ When chromophores, molecules that absorb and emit light, are covalently linked to peptides, the resulting assemblies acquire both optical and structural roles.^105^ This combination enables the engineering of photonic NMs with versatile properties, including tunable spectral features,^106^ anisotropic emission,^107^ and nonlinear optical responses.^108^ These features position chromopeptides as promising candidates for use in photonic crystals, waveguides, and light-harvesting platforms.^109^ Crucially, the peptide sequence and hydrophobicity can be modulated to control the spacing and orientation of chromophores, thereby adjusting electronic coupling and optical bandgaps. Such molecular-level tunability is essential for the development of next-generation photonic devices. One particularly promising characteristic of chromopeptides is their responsiveness to environmental stimuli. Variations in pH, temperature, or ionic strength can alter their optical signatures, such as fluorescence and refractive index, making them well-suited for biosensing and real-time monitoring applications.^110^ Beyond sensing, these nanoarchitectures also offer significant potential for energy management by mimicking natural photosynthetic processes. Their ability to facilitate Förster resonance energy transfer (FRET) and exciton migration opens new avenues for artificial photosynthesis and related light-driven technologies.^20^ Despite these promising attributes, several challenges persist. Key among them is achieving long-range structural order, enhancing quantum efficiency, and maintaining stability under operational conditions. Overcoming these hurdles will require integrated efforts across disciplines such as peptide chemistry, material science, and photonics. Advanced tools, including computational modelling and high-resolution characterization, will be critical to unlocking the full potential of chromopeptide nanoarchitectures.^111^

## 3 Innovating nanomedicine for a healthier tomorrow

Nanomedicine is a growing field which includes imaging, drug delivery, diagnosis, vaccine development, nanoscale scaffolds for tissue engineering and it has obtained consistent investments both from private and public authorities.^112^ Around 80 nano-based pharmaceutical products have been approved by EMA and FDA since 1989.^113,114^ Most of these approved products are organic particles, namely nanocrystals, lipid-based NPs, polymer-based NPs, dendrimer based NPs, protein based NPs, while only 9 products are based on inorganic NPs, of which 6 to 9 are iron NPs for iron deficiency treatment.^115^ More recent advances in this field are biologically derived approaches, such as the use of extracellular vesicles or the development of smart nanocarriers which exploit the function of living cells, harnessing their natural functions for targeted delivery, therapeutic modulation, and improved biocompatibility. However, inorganic NPs may play a crucial role in areas where other approaches fall short, such as the development of novel antimicrobial systems, the development of biosensors, medical and industrial imaging, and as active transducers in photodynamic therapies for cancer treatment. In this section we report several cutting-edge examples of nanomedicine applications, from precision delivery to disease treatment and health monitoring, focusing in drug delivery, innovative treatments, disease detection, the role of microfluidics and further technological applications.

### 3.1 Nanoformulations for pulmonary drug delivery

The emergence of the COVID-19 pandemic incentivized the rapid development and implementation of nanoformulations, particularly as innovative platforms for vaccine delivery and antiviral therapeutics. The success of COVID-19 vaccines highlighted nanotechnology's role as a versatile and promising approach for both preventive medicine and the therapeutic management of infectious diseases.^116–118^ Recently, pulmonary diseases have gained increasing prominence in biomedical research, not only because of the impact of the pandemic, but also due to the global burden of lung cancer, cystic fibrosis and bacterial infections.^119–124^ Conventional treatments for respiratory disorders are mainly administered systemically, often requiring high doses to achieve effective local drug concentration. However, systemic administration can cause off-target effects and systemic toxicity, limiting therapeutic efficacy and patient compliance.^125^ Yet, pulmonary and nasal routes of administration for nanomedicines represent a great opportunity to decrease the drug administrated dose, increase the local concentration on the disease site and decreasing the healthy organs exposure.^122,123,126,127^

**Fig. 4:**
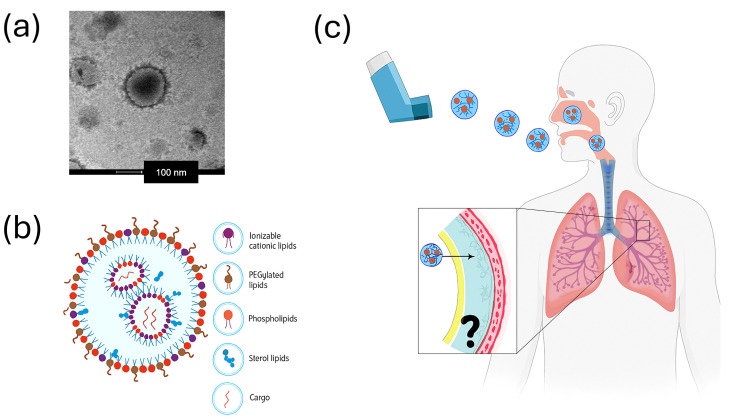
(a) TEM image of lipid NPs used in the mRNA-1273 platform developed by Moderna (Cambridge, MA, USA). Reproduced from Fongaro *et al.*^142^ with permission from Elsevier, Copyright (2023). (b) Schematic of LNP structure composed of lipids encapsulating therapeutic cargo. (c) Illustration of inhalable NPs for nasal, lung or systemic administration, highlighting a key challenge: how different formulations interact with pulmonary fluids like mucus and surfactants as well as macrophages, influencing the uptake.

The lungs, together with the heart, are vital organs working in close coordination to ensure oxygenation of the body through blood circulation. The first ensures the gas exchanges between air and blood, while the second is the pump allowing the blood to circulate in the body. The key to this natural connection between the environment and the systemic circulation is the high vascularization of the lungs as well as the alveoli thin epithelium, also called the air–blood barrier, and the direct proximity of the heart with lungs.^128–130^ Not only can gas cross the air–blood barrier, but studies have also shown that nano- and ultrafine pollution particles can reach the heart *via* oxygenated blood from the lungs, as well as inhaled NMs.^131,132^ This gateway to systemic circulation can be turned into an advantage for medical research, offering a strategic entry point for delivering nanomedicines to the body and particularly for targeting the heart.^130,133^ Traditional drug delivery routes face significant challenges: oral administration is hindered by gastrointestinal barriers, while intravenous or intramyocardial injections are invasive and carry risks of side effects and myocardial injury.^128,129^ Although intranasal administration provides a more direct route than systemic administration, it still requires passage through multiple anatomical barriers and defence mechanisms within the respiratory tract.^134^ NPs offer a promising strategy to protect and selectively deliver drugs to pulmonary tissues, enhancing therapeutic outcomes. The lungs' large surface area and rich blood supply enable the absorption of NPs, allowing fast drug accumulation, with reduced systemic exposure and fewer side effects^129,135^ (Fig. 4).

NPs can vectorize both conventional drugs and biomolecules such as siRNA, miRNA, and other nucleic acids, which are otherwise unstable and difficult to administer.^136^ Nanocarriers protect these labile agents from degradation, permitting their therapeutic use in both infectious diseases and genetic disorders. Additionally, functionalizing NPs with specific proteins or ligands facilitates targeted interaction and drug release at specific cells or tissues, advancing personalized medicine that addresses individual patient needs.^137,138^ Aside from pulmonary diseases, current treatments for heart diseases suffer from poor cardiac specificity and low bioavailability. They only tend to accumulate slightly in cardiac tissue and exhibit short retention times, requiring high doses that lead to toxicity and side effects. Designing and administering inhaled nanomedicines could help overcome these limitations, offering a more targeted, efficient, and safer therapeutic strategy for cardiovascular diseases.^128,129^ Indeed, nanomedicines offer unique physicochemical properties, compatible with alveolar deposition and uptake through the air–blood barrier. Their surfaces can be functionalized, for example, with cardiac-targeting peptides to enhance heart-specific accumulation.^128,129,131,133^ Several studies are currently exploring this strategy, using polymeric, liposomal, inorganic, and especially calcium-phosphate NPs, which are currently among the most studied.^128,131,139^

Looking ahead, future research is focused on the development of smart nanocarriers capable of recognizing biochemical changes, cellular alterations, or tumour markers, and consequently triggering targeted drug release.^140^ In this context, careful selection of NP composition is essential to ensure biocompatibility and avoid pulmonary toxicity. Inhaled nanomedicine represents a promising approach to overcome the limitations of current cardiovascular therapies by improving specificity, efficacy, and safety. Nanotechnology presents vast opportunities in the treatment of pulmonary diseases; however, these are accompanied by significant challenges and unanswered questions that must be addressed to ensure both safety and efficacy.^141^ It is crucial to understand how NPs interact within complex biological systems to avoid their recognition as harmful foreign materials or undesired accumulation that could trigger inflammatory or degenerative responses. Therefore, the development of new nanoformulations should be carried out by multidisciplinary teams capable of thoroughly characterizing NPs from both physicochemical and biological perspectives.

### 3.2 Smart nanocarriers and macrophages for precision medicine

Advances in technology and medicine generated the ‘smart’ nanocarriers, able to bypass physiological barriers with high accuracy, selectivity, and sensitivity. They can exploit the unique characteristics of the biological environment, targeting specific factors involved in the onset and progression of diseases, such as macrophages, and releasing drugs in a controlled manner.^143,144^

Macrophages are immune cells that play a crucial role in surveillance, inflammation, and tissue repair. They are part of the mononuclear phagocyte system (MPS), which also includes cells like monocytes. Known for their ability to ingest foreign material, including pathogens, debris, and NPs, macrophages represent one of the major challenges in nanomedicine.^145^ When nanocarriers are introduced into the organism, these cells often recognize and engulf them through phagocytosis. The clearance can reduce their effectiveness and prevent them from reaching targeted areas, such as tumors or inflamed tissue.^146^ This limitation may present an opportunity in precision medicine.

**Fig. 5:**
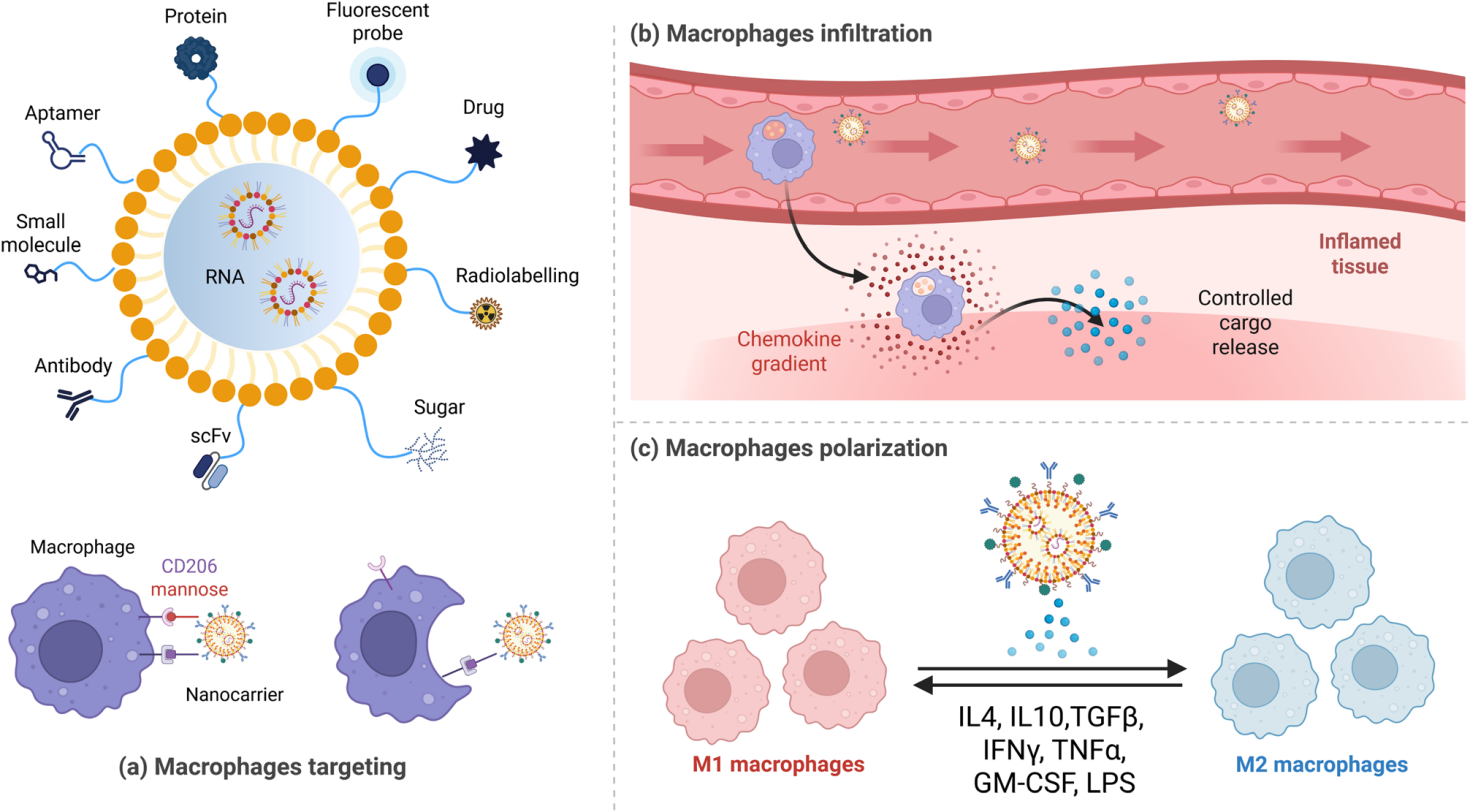
Schematic representation of a nanocarrier-based strategy for macrophage-targeting and immunomodulation. (a) Functionalization of a nanocarrier with different ligands for macrophage targeting. (b) Infiltration of nanocarrier-loaded macrophages into inflamed tissue, enabling controlled release of the therapeutic payload only at the diseased site. (c) Modulation of macrophage polarization *via* nanocarrier-mediated delivery of immunoregulatory agents to promote either pro-inflammatory (M1) or anti-inflammatory (M2) phenotypes. Created with BioRender.com (https://www.biorender.com/).

For instance, macrophages can be targeted as delivery vehicles for nanocarriers, releasing therapeutic agents directly to disease sites during their infiltration. Another strategy is represented by macrophage polarization into the M1 phenotype to promote immune activation against diseases like cancer, or into the M2 phenotype for promoting tissue repair and wound healing in conditions like inflammatory diseases.^147^ They are designed with surface modifications (like ligands or antibodies) that recognize specific receptors on target cells. When these nanocarriers reach their target, they release the cargo in response to specific stimuli (pH, temperature, light, or enzyme activity), ensuring that the drug acts only at the intended site, reducing systemic side effects.^148^ Smart nanocarriers can not only deliver drugs, but also agents that modulate macrophage activity, promoting the desired immune response.^149^ An emerging strategy involves using RNA molecules to treat genetic disorders and cancer. Nanocarriers can be used to deliver these unstable molecules to cells, including macrophages, where they can modulate gene expression to reprogram immune responses or fight disease^150^ (Fig. 5). Their unique properties and versatility make them applicable in various biomedical fields, for treating cancer,^149^ chronic inflammation,^151^ neurological disorders,^152^ and promoting tissue regeneration.^153^

Smart nanocarriers hold potential for precision medicine. By exploiting macrophages' natural functions, these systems enhance drug delivery, modulate immune responses, and provide effective treatments for various disorders.^154^ The integration of artificial intelligence and machine learning could allow the design of more responsive, adaptive nanocarriers specific for individual patient profiles. Moreover, advances in RNA therapeutics and gene editing may lead to even more precise modulation of macrophage function, unlocking new possibilities for treating genetic and immune-related disorders. Innovative delivery platforms capable of multi-functional payload release, real-time *in vivo* monitoring, or self-regulating therapeutic responses are emerging. As research improves and interdisciplinary collaborations grow, smart nanocarriers may become central to the next generation of personalized therapies.

### 3.3 Extracellular vesicles as a next-generation therapeutic platform

Extracellular vesicles (EVs) are membrane-bound NPs naturally released by cells that carry proteins, lipids, and nucleic acids from their cell of origin. Once thought to be cellular waste, EVs are now recognized as key mediators of intercellular communication, and interest in their therapeutic potential has grown rapidly.^155^ Their role in diverse physiological and pathological processes, including cancer, neurodegeneration, and inflammation, has made EVs attractive as both biomarkers and therapeutic delivery systems. Several characteristics make EVs especially well-suited for drug delivery: they are stable in circulation, can naturally target specific cell types, and can be engineered to deliver a variety of therapeutic molecules, from small RNAs to proteins and small-molecule drugs.^156,157^ Furthermore, unlike synthetic NPs, EVs have an inherent ability to evade immune detection, cross biological barriers (including the blood–brain barrier), and deliver complex bioactive molecules to target tissues.^158^ And because they are derived from cells, they offer a level of biocompatibility and safety that is hard to match with current delivery systems. Their potential uses are remarkably broad: from treating cancer (by delivering drugs or serving as cancer vaccines), to regenerating injured tissues (by delivering growth factors or RNAs), to modulating the immune system in autoimmune diseases, to even crossing into the brain to treat neurological conditions.

Nevertheless, despite rapid progress, several hurdles remain before EV therapies can become mainstream. Scalable production is a major bottleneck: current isolation methods are not easily adapted to clinical-grade, large-scale manufacturing. EV heterogeneity also complicates characterization and quality control, making it difficult to link therapeutic effects to specific cargo. The field still lacks standardized protocols for isolation, dosing, and potency assessment, though guidelines like MISEV2023 are helping address this.^159^ Regulatory frameworks are also evolving; EVs do not fit neatly into existing drug categories, and approval pathways remain unclear. Finally, biodistribution and targeting remain challenging, with many EVs accumulating in off-target organs. Addressing these challenges through better analytics, standardization, and regulatory dialogue will be essential to advancing EVs as a clinically validated delivery platform.

Even so, progress is well underway, and in recent years EV-based therapeutics have begun to move from the bench to the clinic. Most preclinical studies have focused on inflammation, cancer, tissue regeneration, and neurological disorders, where EVs, often from stem or immune cells, are used to deliver bioactive molecules. Clinical translation, however, remains in its early stages. As of 2024, only a few dozen clinical trials have tested EV-based therapies in humans.^160^ These have mostly been small-scale pilot studies, with wide variation in disease targets, EV sources, doses, and administration routes – making it difficult to draw sound conclusions or compare outcomes across trials. Still, initial results are promising: a recent systematic review and meta-analysis reported a very low incidence of serious adverse events (∼0.7%), supporting the idea that EVs are generally safe.^161^ Such outcomes, while preliminary, reinforce the view that EVs hold genuine therapeutic promise. In the coming years, larger Phase II/III trails with more uniform protocols will be key to determining how EVs are effective across different diseases.

### 3.4 Nanomaterials as broad-spectrum antimicrobial alternatives in the fight against infectious diseases

The increasing prevalence of infectious diseases, coupled with the accelerating threat of antimicrobial resistance (AMR), has prompted urgent global efforts to identify effective alternatives to conventional antibiotics. The therapeutic efficacy of many frontline antibiotics is rapidly diminishing, and clinicians are often left with limited options to contain the spread of resistant pathogens. In this context, engineered NMs have emerged as promising broad-spectrum antimicrobial agents with versatile applicability beyond the scope of traditional antibiotics.^162^ Both organic and inorganic NPs exhibit antimicrobial properties, albeit with distinct advantages and limitations. Organic nanomaterials, most notably CBNs, are primarily valued for their high biocompatibility, structural versatility, and strong potential as carrier platforms, while still expressing intrinsic antimicrobial activity. In addition, their biodegradability is considered advantageous in mitigating long-term environmental concerns. However, for applications outside of biological systems, industrial development has predominantly focused on inorganic NPs. A key reason for this preference is the central role of ion release as a highly potent antimicrobial mode of action, which is characteristic of many inorganic NMs but is not a dominant mechanism in organic nanomaterials.^163^ Metallic and metal oxide NPs, in particular, exhibit intrinsic antimicrobial properties through multiple mechanisms of action, including ion release, disruption of microbial membranes, and the generation of reactive oxygen species (ROS).^164^ These effects depend primarily on three factors: (i) the chemical composition of the NM, (ii) its physicochemical properties (*e.g.*, size, surface charge, solubility^165^), and (iii) surface modifications or functionalizations.^166^ Such multifaceted mechanisms reduce the likelihood of resistance development and can be readily tailored by modifying NP characteristics, enabling precise design strategies for targeted applications. A key advantage of NMs over conventional antibiotics lies in their broad-spectrum activity. While antibiotics are typically species-specific and limited to bacterial infections, NPs such as ZnO, Ag, and CuO have demonstrated efficacy against a wide range of pathogens, including bacteria, fungi, and viruses.^167^ This expanded spectrum of activity makes them particularly attractive as preventive agents in both clinical and non-clinical environments. Given their tunability, NMs are increasingly being considered for incorporation into everyday objects to mitigate the spread of infections. Potential applications include embedding antimicrobial coatings into high-contact surfaces such as mobile phones, keyboards, handrails, and doorknobs, where pathogen transmission is common. In healthcare settings, surfaces of medical devices and workstations could be coated with biocidal NMs to minimize nosocomial infections. The scalability and cost-effectiveness of such preventive measures could significantly reduce the incidence of infectious diseases in public and clinical environments. Beyond healthcare and consumer safety, NMs are also being explored in agricultural and environmental contexts. For example, nano-enabled crop coatings could reduce fungal infestations and mitigate crop losses, while the integration of antimicrobial NMs into wastewater treatment systems offers a route to inactivate pathogenic organisms and improve sanitation.^168^ Therefore, antimicrobial NMs represent a versatile and potent platform with far-reaching potential to combat pathogens across sectors. Their unique physicochemical properties, tunable antimicrobial activity, and broad applicability position them as promising candidates in a multi-pronged strategy to address the global challenge of infectious diseases and AMR.

### 3.5 Nanoparticles for radiological theranostic

In the field of medical diagnostics, the detection of ionizing radiation (essential for CT and PET imaging) faces a constant demand for higher sensitivity, better resolution, and easier processability.^169^ These requirements rely critically on scintillators, materials that convert high-energy radiation into visible light for signal detection. However, conventional bulk scintillators often involve trade-offs between performance, cost, and fabrication complexity.^170^ Similarly, in Photodynamic Therapy (PDT), limited light penetration hinders the treatment of deep-seated tumors. Nanomaterials address these challenges through X-ray Photodynamic Therapy (X-PDT), which utilizes X-rays to reach deep tissues.^171^

Unlike bulk scintillators, which require expensive high-temperature procedures like the Czochralski method and suffer from light scattering, nanoscintillators are produced *via* cost-effective wet chemical methods.^170^ Their nanoscale nature enables tunable emissions (from UV to NIR-II) and custom core–shell designs to optimize performance.^172^ Among tunable luminescent platforms, the NaREF_4_ (RE = rare-earth element) system stands out as an example of structural and optical customization. Furthermore, quantum confinement effects allow for enhanced light yield and ultrafast scintillation kinetics, with decay times reaching the sub-nanosecond regime^173^ due to modified electronic relaxation processes.^174^

Nanoscintillators also improve spatial resolution: while excited regions in bulk materials extend over tens of micrometers, nanoscintillators reduce this to a few micrometers, allowing more precise localization of scintillation events.^174^ Additionally, heterostructured approaches combine complementary properties—such as high atomic number for attenuation and fast response for timing—to optimize detector performance.^173^

In X-PDT, scintillating nanoparticles act as internal light sources, bridging the gap between deep X-ray penetration and photosensitizer activation.^175^ High-Z elements within these particles, such as LnF_3_, amplify X-ray absorption and ROS generation.^176^ This enhancement allows effective tumor control with significantly lower radiation doses (*e.g.*, 4 Gy with nanocarriers *vs.* 12 Gy for radiotherapy alone), reducing systemic side effects.

Nanocarriers further improve photosensitizer stability and delivery through the enhanced permeability and retention (EPR) effect or active targeting with specific ligands.^176^ Encapsulation ensures controlled release (a remarkable example are Gd_2_O_2_S:Tb nanocapsules) and protects agents from degradation.^177^ Finally, incorporating oxygen-generating nanoagents (*e.g.*, catalase-loaded materials) addresses tumor hypoxia.^178^ Collectively, nanocarriers transform X-PDT into a high-precision modality, maximizing therapeutic impact while minimizing toxicity.

### 3.6 Nanomaterials for biosensors monitoring of health

The increasing research interest in personalized medicine has led wearable sensors to be great candidates to improve health care and continuous control of the quality of life, giving information about the effectiveness of the care, an early detection of disease and real-time monitoring.^179,180^ Since wearable biosensors must be selective and sensible, NMs are a good choice to enhance interaction with biological targets, thanks to their high surface-to-volume ratio, improving sensitivity in the detection.

A wide range of NMs – including carbon-based structures (graphene,^181^ graphene oxide,^182^ carbon nanotubes,^183^ fullerene^184^), metal and metal oxide NPs (gold,^185^ platinum,^186^ silver,^187^ copper^188^), and metal–organic frameworks^189^ – has been integrated into wearable devices. For instance, graphene-based sensors have demonstrated high sensitivity and flexibility, making them ideal for integration into epidermal patches^190^ and smart textiles.^191^ Gold NPs and quantum dots are widely used for optical sensing due to their tunable fluorescence and plasmonic properties.^192^

**Fig. 6:**
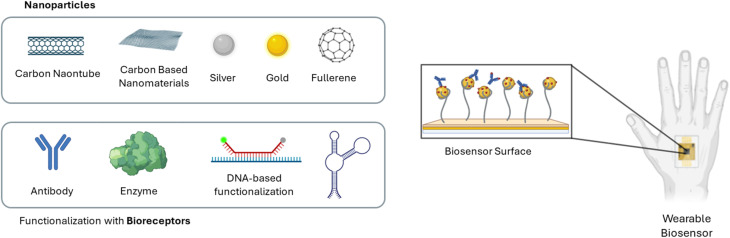
Schematic illustration of wearable biosensors based on nanomaterials and their functionalization with common bioreceptors. Created with BioRender.com (https://www.biorender.com/).

Moreover, the functionalization^193^ of NMs with biomolecules (*e.g.*, enzymes, aptamers, antibodies, DNAzyme^194^) allows for high specificity toward target analytes, enabling personalized and precision medicine approaches (Fig. 6). Recent advances in nanofabrication^195^ and microfluidics^196^ have also facilitated the development of multiplexed platforms capable of monitoring multiple biomarkers simultaneously, improving the accuracy and reliability of health assessments.

Despite these advancements, several challenges remain before large-scale clinical translation can be achieved.^197–199^ These include long-term biocompatibility, sustainability and device stability. Nonetheless, the integration of NMs with wireless communication systems and artificial intelligence^200^ is opening new avenues for continuous and remote patient monitoring, with the potential to shift healthcare from reactive to proactive models.

### 3.7 Microfluidics in drug delivery: bridging precision and innovation

**Fig. 7:**
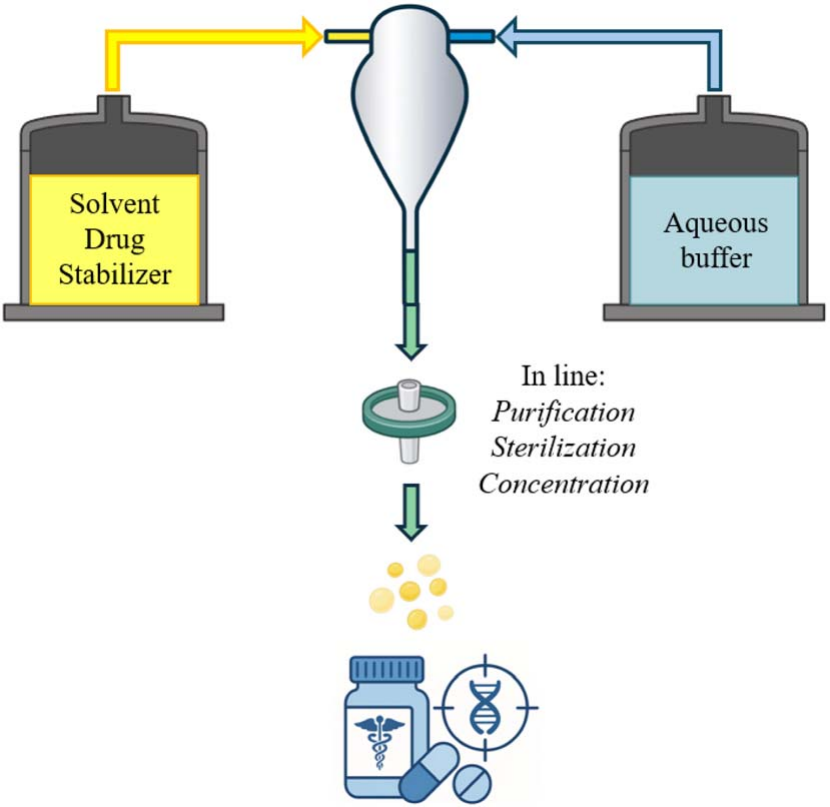
Schematic representation of nanoparticle preparation for drug delivery using an impingement jet mixer.

Microfluidics is a technology that enables precise manipulation and control of fluid flow within channels characterized by at least one dimension smaller than 1 µm. This miniaturization allows access to fluidic behaviours not observable at the macroscale, making microfluidics a highly versatile platform. It is widely applied across diverse disciplines, including fluid dynamics, synthetic and analytical chemistry, biology, and medicine. Key applications include drug toxicity assessment, the development and characterization of drug delivery systems, regenerative medicine, and single-cell analysis.^201^ Microfluidics presents significant advantages in the development of drug delivery systems (DDS), primarily due to its miniaturized scale, which reduces material consumption. Its precise control over experimental conditions enables the fabrication of particles with clinically relevant properties, eliminating the need for additional post-production processing (such as extrusion) reducing the risk of cargo unloading or degradation.^202^ Furthermore, microfluidic platforms allow for the rapid and systematic testing of multiple parameters with processes that are inherently scale-independent. Therefore, microfluidics enables rapid optimization of DDS characteristics using a design-of-experiments approach, followed by the seamless translation of formulation and processing parameters from laboratory development to industrial scale with a continuous process. Continuous manufacturing offers several general advantages, including reduced capital expenditure, a smaller manufacturing footprint, and lower cost of goods. In the context of DDS development, continuous manufacturing enables the integration of critical processing steps such as purification, concentration adjustment, and sterilization, directly within the continuous flow, enhancing efficiency and process control.^203^ These advantages have been proved by Pfizer's mRNA COVID-19 vaccine production. Indeed, the vaccine's lipid NPs, encapsulating the mRNA, were produced using an impingement jet mixer (IJM). IJMs are passive mixing devices, firstly reported in 2003,^204^ known for their ability to achieve rapid and efficient mixing at microscale dimensions while enabling high throughput^205^ (schematic workflow in Fig. 7). Instead of scaling up mixer size, Pfizer scaled out by parallelizing 100 IJMs, enabling continuous synthesis and achieving a production rate of 100 million doses per month. Automation ensured precise control over flow and pressure.^32^ This approach demonstrated the scalability, efficiency, and versatility of microfluidics in producing nanomedicines for large-scale drug delivery. Various approaches can be employed to develop DDS using microfluidics, primarily differing in the method of flow mixing used to induce particle self-assembly. Microfluidic technologies for DDS development have largely focused on the production of organic NPs, particularly lipid-based nanomedicines. This class of NPs has seen substantial growth, as evidenced by a rising number of clinical trials and FDA approvals and now represents a central component of modern therapeutic strategies.^206^ New microfluidic systems are continually being developed to produce DDS, increasingly leveraging advancements in 3D printing technologies. However, these innovations to reach industrial application, must undergo rigorous regulatory evaluation and receive approval from agencies such as the European Medicines Agency (EMA) or the Food and Drug Administration (FDA). This process, particularly for novel microfluidic devices, demands extensive clinical testing and demonstration of long-term safety and efficacy, which can be both time-consuming and costly.^207^ Conversely, commercially available off-the-shelf microfluidic kits, designed with scalability in mind, offer faster route to therapeutic development. Their production flexibility makes them especially valuable for personalized medicine, enabling patient-specific dosing and rapid adaptation to individual treatment needs.^208^

## 4 Nanomaterial characterization and ethical testing for responsible nanosafety assessment

NMs are expected to play a central role in the transition to greener technologies and improved healthcare, but their full potential remains limited by the lack of practical, standardized methods for evaluating their safety and interactions in complex systems. Advancing nanosafety demands strategies that reflect how NMs truly behave in relevant biological and environmental media. The future lies in developing new protocols that capture dynamic transformations, and realistic exposure profiles, while embracing animal-free, high-throughput models such as *C. elegans* in line with the 3Rs (Replacement, Reduction, and Refinement) principles.^209^ Integrating these approaches will provide more predictive, ethical, and standardized assessments, ensuring safer design and deployment of next-generation NMs but also proper assessment of impact of emerging NM pollutants.^210^

### 4.1 Advancing characterization from standard nanomaterials to industrially relevant and environmental contaminants

A major challenge in nanosafety assessment lies in the insufficient characterization of these materials under realistic exposure conditions, especially when tested in biological or environmental media. Once introduced into such media, NMs undergo dynamic physicochemical transformations that alter their size, aggregation state, surface properties, and most importantly, lead to the formation of a biomolecular corona.^211^ This corona is a layer of proteins, lipids, and other biomolecules that adsorbs onto the NM surface and it fundamentally redefines the biological identity of the particle and governs its interactions with cells and tissues.^212^ Significant efforts have been done to mitigating these transformations through surface functionalization with biocompatible polymers,^213^ such as poly(ethylene glycol) (PEG),^214–216^ poly(ethylenimine) (PEI),^217^ poly(amidoamine) (PAMAM) dendrimers,^218,219^ chitosan,^220,221^ and related coatings. While these modifications improve colloidal stability and quench biomolecular corona formation, even functionalized nanomaterials undergo dynamic transformations under realistic biological and environmental conditions.^222^ And despite its importance, the corona is still often overlooked in *in vitro* studies, particularly in standard toxicity tests that do not consider the species origin of serum components or the effect of preconditioning protocols.^223,224^ Therefore, a thorough characterization of the corona, especially the hard corona that persists and dictates interactions,^225^ is essential for reliable hazard assessment. In particular beside proteomic, the workhorse of corona characterization, the role of other biomolecules is emerging such as glycans or lipids.^226,227^ Moreover, it is also important to be aware of the advantages and limits of the established protocols such as for proteomics analysis.^228^ In addition to corona profiling, the colloidal stability and state of dispersion of NMs in test media are critical factors.^147,229^ Many NMs, such as graphene or some kind of quantum dots, are not initially designed to be water-dispersible and often require mechanical or chemical dispersion to be evaluated *in vitro*.^230^ Improper dispersion leads to uncontrolled agglomeration and sedimentation, which in turn results in inaccurate dose delivery.^231^ However, dosimetry remains one of the most underappreciated yet impactful factors in nanotoxicology. Indeed, the actual dose delivered to cells is not simply the concentration administered but a function of time-dependent transformations such as agglomeration, sedimentation, and diffusion, all of which are influenced by particle size, shape, surface chemistry, and effective density. Large agglomerates sediment faster, increasing the local concentration at the cell surface, while buoyant particles may float away, preventing contact entirely. Moreover, agglomeration decreases the number of bioavailable particles and reduces total surface area, a key determinant of nano–bio interactions. Accurate dosimetric modeling, incorporating these transformations and validated by *in vitro* to *in vivo* extrapolation, is crucial to resolving the discrepancies that frequently arise between.^231^ The importance of the assessment of the real delivered dose is also fundamental for low-density materials such as plastics like polyethylene and polypropylene, which are buoyant and do not settle onto the cell layer in traditional assays. These materials, while poorly represented in current studies, are the most abundant in human exposure scenarios, including ingestion and environmental contamination.^232–234^ Their irregular shapes, broad size distributions, and resistance to aqueous dispersion present unique challenges for both characterization and testing. To capture realistic exposure profiles, specialized models such as inverse cell culture systems are necessary, especially for floating particles.^235^ Without such adaptations, results may systematically underestimate or misrepresent toxicity.

In conclusion, as the diversity of NMs increases, with the emergence of complex composites, 2D materials with high surface areas, non-aqueous dispersible particles, and environmentally derived NMPs, there is an urgent need for robust and adaptable characterization strategies. These materials often exhibit unique behaviours that are not adequately captured by standard protocols.^230,236^ Their heterogeneity, non-spherical morphology, and interactions with environmental or biological macromolecules challenge conventional assumptions and necessitate the use of orthogonal techniques to fully assess size, aggregation, surface chemistry, and interaction potential in relevant media.

### 4.2 Influence of surface charge of plastic nanomaterials on interaction with biological models

**Fig. 8:**
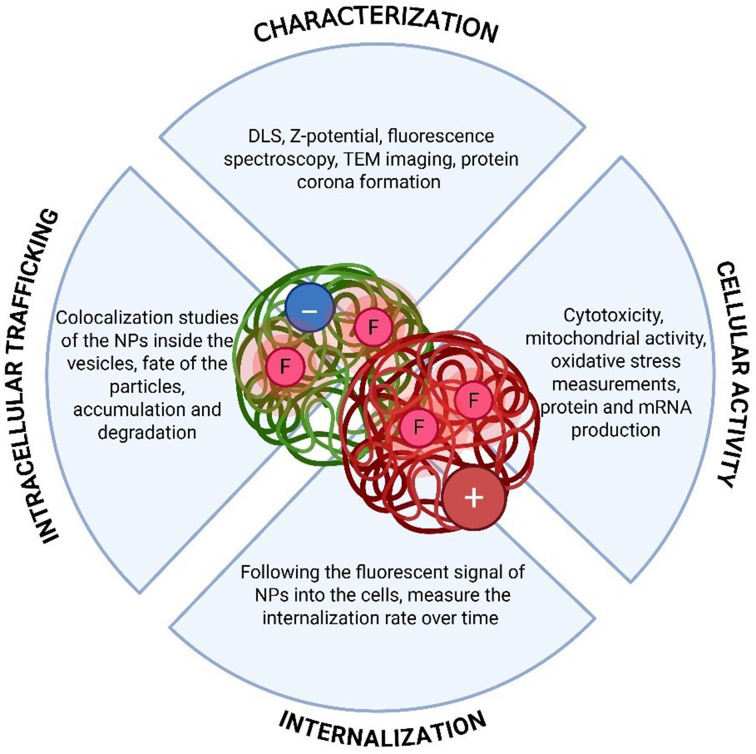
Development of a platform suitable for different NMs, to study features of NPs-biological targets interaction.

Plastic industry is one of the main revolutions of the last century. Production of plastic has increased more than 100% per year.^232^ Also, the COVID-19 pandemic has further increased the demand for single-use plastic materials for daily usage.^237^ Although the effect of macroplastics has been extensively investigated,^238,239^ the interaction between micro and nanoplastics and biological targets needs further clarifications.^240,241^ Understanding how NP features, such as the external surface charge, impact the uptake by cells, the intracellular fate and, on a larger scale, the environment and health is needed to improve sustainable plastics production. To this aim, an integrated platform including physical and chemical characterization, cellular activity studies, internalization and intracellular path and fate of NPs, also suitable and tunable for many different NMs, has been developed (Fig. 8).

Human cell lines and *C. elegans* are biological models commonly employed to study the cytotoxicity of various materials. Recent studies revealed that positive and negative polystyrene nanoplastics (PS-NPs) act differently in the interaction with cells and animal models. Positively charged NPs tend to be more internalized than negatively charged ones, also causing a dose-dependent decrease in cell growth and viability. The *z*-potential of PS-NPs also affected their toxic effect *in vivo*, where only positive NPs are able to cause a dose-related decrease of *C. elegans* viability and defects in motility, pharyngeal function, reproduction, and development. Investigating the route through which the NPs enter the cells result crucial to better understand their fate once internalized. Latest findings confirmed that cells primarily exploit clathrin-mediated endocytosis to uptake PS-NPs, depending also on the NPs size and *z*-potential.^242^ In particular, highly positively charged NMs, get attached to the negative charge of the cell membrane because of electrostatic attraction and then penetrate the membranes. For the same reason, a charge repulsion between negative PS-NPs and the cell membrane could lead to a minor internalization rate. Furthermore, the surface charge of the PS-NPs impacts the formation and the composition of the protein corona once the particles interact with biological fluids. Experiments based on the incubation of PS-NPs with serum show a lower rate of internalization of both positively and negatively charged PS-NPs into cells, broadening the research field of the protein corona.^243^

The concern about these nanometric particles is their fate once internalized into the cells. Usually, when internalized, NPs are rapidly entrapped into the early endosomes starting the degradation path through the late endosomes and lysosomes. However, some particles including ionizable lipid NPs (such as mRNA based nanovaccines) are able to escape from the endosomes, through the endosomal escape, and release their cargo into the cytoplasm.^142^ Nanomedicine often takes advantage of this feature for its therapeutic aims. In the case of PS-NPs, the issue could be related to the long-term effects of the accumulation in various districts of our body. Thus, the evidence that after 24 hours a high percentage of NPs are already entrapped into the lysosomes suggest an efficient degradation of the particles. The effect of the accumulated particles over time is still unknown and deserves a deeper investigation, for human, animal and environmental health.

### 4.3 *C. elegans* as an *in vivo* model for toxicological studies on nanomaterials

*Caenorhabditis elegans* (*C. elegans*) is a free-living, non-parasitic nematode that is approximately 1 mm in length and is widely used as a model organism in biomedical research. It has a well-characterized anatomy consisting of around 1000 somatic cells, including a fully mapped nervous system with 302 neurons.^244^ Despite being evolutionarily distant from vertebrates, 65% of its genes have a homolog in humans, and many pathways involved in development, cell death, metabolism, and stress responses are conserved.^245^*C. elegans* offers several technical advantages: a short lifecycle, rapid reproduction, a transparent body for *in vivo* imaging, and well-established genetic manipulation techniques, such as RNAi and the development of transgenic worms.^245,246^ These characteristics make it a valuable tool for toxicological studies, particularly in the context of NMs.

**Fig. 9:**
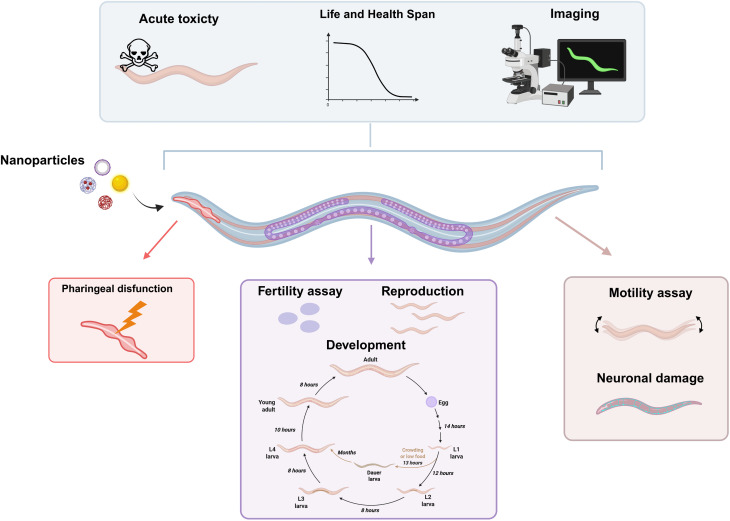
*Caenorhabditis elegans* as a model to assess nanoparticle toxicity. The figure illustrates some endpoints that can be evaluated using this model, including acute and long-term toxicity, localization and expression of fluorescent molecules, pharyngeal function, fertility, reproduction, development, motility, and neuromuscular damage. Created with BioRender.com (https://www.biorender.com/).

Exposure studies can be conducted *via* ingestion, dermal absorption, or environmental exposure in liquid or agar-based media. Key endpoints include lethality, reproductive output, developmental progression, behavioural phenotypes, and molecular biomarkers such as oxidative stress genes or heat-shock proteins^246,247^ (Fig. 9). *C. elegans* is also helpful in assessing the chronic effects of low-dose NM exposure. Together, this application renders *C. elegans* a valuable model for high-throughput assays.^248^

Another advantage of using *C. elegans* is that this nematode is ubiquitous worldwide.^249^ It inhabits both soil and water at varying temperatures, thus serving as an excellent environmental indicator that provides insight into the impact of pollution on the health of living organisms. Ultimately, *C. elegans* offers a biologically relevant and ethically advantageous alternative to vertebrate models, aligning with the principles of the 3Rs and facilitating the early-phase screening of NM toxicology, thereby promoting the safe development of new NMs.

Several studies have been published using *C. elegans* as an animal model to evaluate the toxicity of various NMs.^243,247,250,251^ For instance, the relationship between the physicochemical properties of PS-NPs, particularly the surface charge, and their toxicity *in vitro* and *in vivo* was recently studied. The *in vivo* findings obtained from *C. elegans* supported those acquired from cells, providing further insights into the sub-toxic effects of PS-NPs, which are impossible to detect in simpler systems, such as cell culture.^243^

Significant challenges persist in the field of nanotoxicity. Chief among these is translating the effects observed in *C. elegans* to human health risk assessment. This nematode lacks certain tissues, such as a circulatory system, making it difficult to fully understand the biodistribution of NMs. Additionally, the absence of organs like the liver, brain, and lungs obstructs the study of organ-specific effects. A clear need exists for collaboration among various laboratories to validate findings from *C. elegans* through studies on more complex vertebrate models. It is essential to develop standardized protocols for the physicochemical characterization of NMs and for assessing their toxicity in *C. elegans*. Generating comparable and reliable data will foster acceptance within the scientific community and among nanotoxicologists regarding the use of *C. elegans* as a model for preclinical analysis of NM toxicity.

In conclusion, looking to a future in which the development of NMs for medical or industrial use takes over, *C. elegans* represents a promising and versatile *in vivo* model for the early-stage toxicological evaluation of NMs, not only for the analysis of classical endpoints, such as survival, reproduction, and locomotion, but also by its integration into high-throughput assay, and from an ethics point of view. However, to fully realize its potential, strengthening collaboration between scientists and harmonizing methodologies will be critical steps toward integrating *C. elegans* into regulatory frameworks and preclinical analysis, ultimately advancing the safe and responsible development of nanomedicines.

## 5 Summary and perspective

This review has explored the rapidly expanding *Nanoverse*, a conceptual framework capturing the diversity and interconnectivity of NMs across disciplines. As reported in this review, these advances collectively illustrate how precision molecular design, hybrid architectures, and scalable manufacturing strategies are enabling NMs to address current biomedical, environmental, and industrial challenges.

At the same time, we have highlighted the critical importance of realistic characterization and ethically responsible toxicological assessment. The behaviour of NMs in complex biological and environmental matrices is governed not only by their intrinsic physicochemical parameters but also by dynamic transformations such as biomolecular corona formation. Understanding these processes, particularly under realistic exposure conditions, remains a prerequisite for accurate risk assessment. In this regard, novel models such as *C. elegans* offer a biologically relevant and ethically sound complement to conventional *in vitro* systems, enabling high-throughput, whole-organism toxicology while aligning with the principles of the 3Rs. We also highlight how this is also crucial, not only to properly assess the impact of manufactured NMs, but also for the study of emerging NM contaminants, such as micro- and nanoplastics. Indeed, these NMs further highlight the need for adaptable protocols capable of addressing materials with irregular morphologies, broad size distributions, and atypical dispersion behaviours.

Despite all the reported advances, the *Nanoverse* is still in its early stages. Major challenges persist, including the absence of universally accepted descriptors, limited cross-domain data integration, and difficulties in scaling promising lab-scale innovations to industrial production. Meeting these challenges will require the integration of advanced computational tools into NM research pipelines. Molecular simulations, from quantum-level modelling of defect chemistry to mesoscale dynamics of corona evolution, can now be coupled with Quantitative Structure–Activity Relationships (QSAR) and machine learning algorithms to predict NM behaviour across multiple environments and time scales.^252–254^ Artificial intelligence (AI) can accelerate discovery by enabling inverse design approaches, identifying optimal structural parameters for targeted functions, and guiding adaptive manufacturing processes.^255–257^ These tools are equally crucial for safety-by-design, allowing the early identification of potential hazards before materials enter biological systems or the environment.

Looking ahead, the evolution of the *Nanoverse* will depend on a synergistic interplay between experimental innovation, computational prediction, and regulatory adaptation. International collaboration will be essential for developing standardized characterization frameworks, ensuring data interoperability, and facilitating the translation of novel NMs into safe, high-performance applications. By merging advances in material science, bioengineering, AI-driven modelling, and nanosafety assessment, we can accelerate the responsible expansion of the *Nanoverse*, unlocking its potential to transform our society while safeguarding human and planetary health.

## Author contributions

A. Sao.: conceptualization, investigation, visualization, project administration, writing – original draft, writing – review & editing; A. Su.: investigation, visualization, writing – original draft, writing – review & editing; G. S.: investigation, visualization, writing – original draft, writing – review & editing; G.-Y. M.: investigation, visualization, writing – original draft, writing – review & editing; C. N.: investigation, visualization, writing – original draft, writing – review & editing; R. B.-P.: investigation, visualization, writing – original draft, writing – review & editing; G. C.: investigation, visualization, writing – original draft, writing – review & editing; M. G.: investigation, visualization, writing – original draft, writing – review & editing; M. C.: investigation, writing – original draft, writing – review & editing; M. V.-B.: investigation, writing – original draft, writing – review & editing; A. Sal.: investigation, writing – original draft, writing – review & editing; B. P.: investigation, writing – original draft, writing – review & editing; S. C.: investigation, writing – original draft, writing – review & editing; A. M.: investigation, visualization, writing – original draft, writing – review & editing; A. M.-S.: conceptualization, supervision, visualization, project administration, writing – original draft, writing – review & editing.

## Conflicts of interest

The authors declare that the research was conducted in the absence of any commercial or financial relationships that could be construed as a potential conflict of interest.

## Data Availability

No primary research results, software or code have been included and no new data were generated or analysed as part of this review.
